# Interobserver agreement between an artificial intelligence algorithm and colon capsule endoscopy readers on bowel-cleansing quality

**DOI:** 10.1016/j.igie.2023.04.006

**Published:** 2023-05-23

**Authors:** Benedicte Schelde-Olesen, Jürgen Herp, Jan-Matthias Braun, Anastasios Koulaouzidis, Thomas Bjørsum-Meyer, Lasse Kaalby, Gunnar Baatrup, Esmaeil S. Nadimi, Ulrik Deding

**Affiliations:** 1 Department of Clinical Research, University of Southern Denmark, Odense, Denmark; 3 Applied AI and Data Science Group, Mærsk-Mc-Kinney Møller Institute, Faculty of Engineering, University of Southern Denmark, Odense, Denmark; 2 Department of Surgery, Odense University Hospital, Svendborg, Denmark; 5 Department of Medicine, Odense University Hospital, Svendborg, Denmark; 4 CAI-X (Centre for Clinical Artificial Intelligence), University of Southern Denmark and Odense University Hospital, Denmark; 6 Department of Social Medicine and Public Health, Pomeranian Medical University, Szczecin, Poland

## Abstract

**Background and Aims:**

Colon capsule endoscopy (CCE) faces substantial challenges, one of which is achieving adequate colon cleansing. Furthermore, the interobserver agreement on bowel-cleansing quality varies. To address this issue, we developed an artificial intelligence algorithm (AIA) to evaluate bowel-cleansing quality. The aim of this study was to estimate the interobserver agreement on bowel cleansing between a group of experienced CCE readers and an AIA and to examine whether percentiles of the overall bowel-cleansing quality are a suitable way of reporting the results generated by the AIA.

**Methods:**

Bowel-cleansing quality in 842 CCE investigations was scored on both a 2- and 4-point grading scale for the entire colon and by segment by experienced CCE readers and the AIA. For the algorithm, a score was given based on the mean score, median, upper and lower quartiles, and second and 98th percentiles. The level of agreement was evaluated using Cohen’s κ.

**Results:**

The interobserver agreement between the CCE readers and AIA on bowel-cleansing quality was minimal to none for the overall bowel evaluation, by segment, and on the 2- and 4- point grading scale regardless of the threshold for the AIA score.

**Conclusions:**

We found minimal agreement on evaluation of bowel-cleansing quality in CCE between CCE readers and the AIA. Mean or percentiles of the AIA grading did not seem suitable for AI-generated bowel-cleansing evaluation.

The primary purpose of endoscopic procedures is to visualize the mucosa and detect colorectal neoplasia. Colon capsule endoscopy (CCE) was introduced in 2006 in Europe as an alternative diagnostic tool for the colorectum.^1^ However, CCE still faces substantial challenges, one of which is achieving adequate bowel cleansing and good-quality visualization.^2^ At conventional colonoscopy, fluid and fecal matter can be, to some extent, aspirated through the scope to improve mucosal visualization and quality of the examination; such intervention is not available during CCE. This leads to additional requirements for bowel preparation regimens to reduce re-examination rates.

Only a few grading scales have been developed specifically for grading bowel-cleansing quality in CCE. The Leighton-Rex scale is the most commonly used.^3^ However, it is a qualitative scale that is reader-dependent and inherently subjective, with high intra- and interobserver variation.^3^, ^4^, ^5^ In addition, completion rates and interobserver agreement on bowel preparation in CCE vary between trials.^3^, ^4^, ^5^, ^6^ By introducing an artificial intelligence algorithm (AIA), we may obtain a more objective and consistent evaluation of the quality of bowel cleansing. Our objectives were to examine the interobserver agreement on bowel-cleansing quality between a group of experienced CCE readers and an AIA and to evaluate whether percentiles of the overall bowel-cleansing quality are a suitable way of reporting the results generated by the AIA.

## Methods

All CCE investigations analyzed in this study were from participants in the Danish colorectal cancer screening program as part of the CareForColon2015 trial, which investigates CCE performance in colorectal cancer screening.^7^^,^^8^ Patients were between 50 and 75 years of age with a positive fecal immunochemical test (≥100 ng hemoglobin/mL buffer).^9^ In the CareForColon2015 trial, CCE videos were evaluated by experienced CCE readers, and CCE completion rates were continuously monitored.

### CCE readers: cleansing quality grading

**Figure 1:**
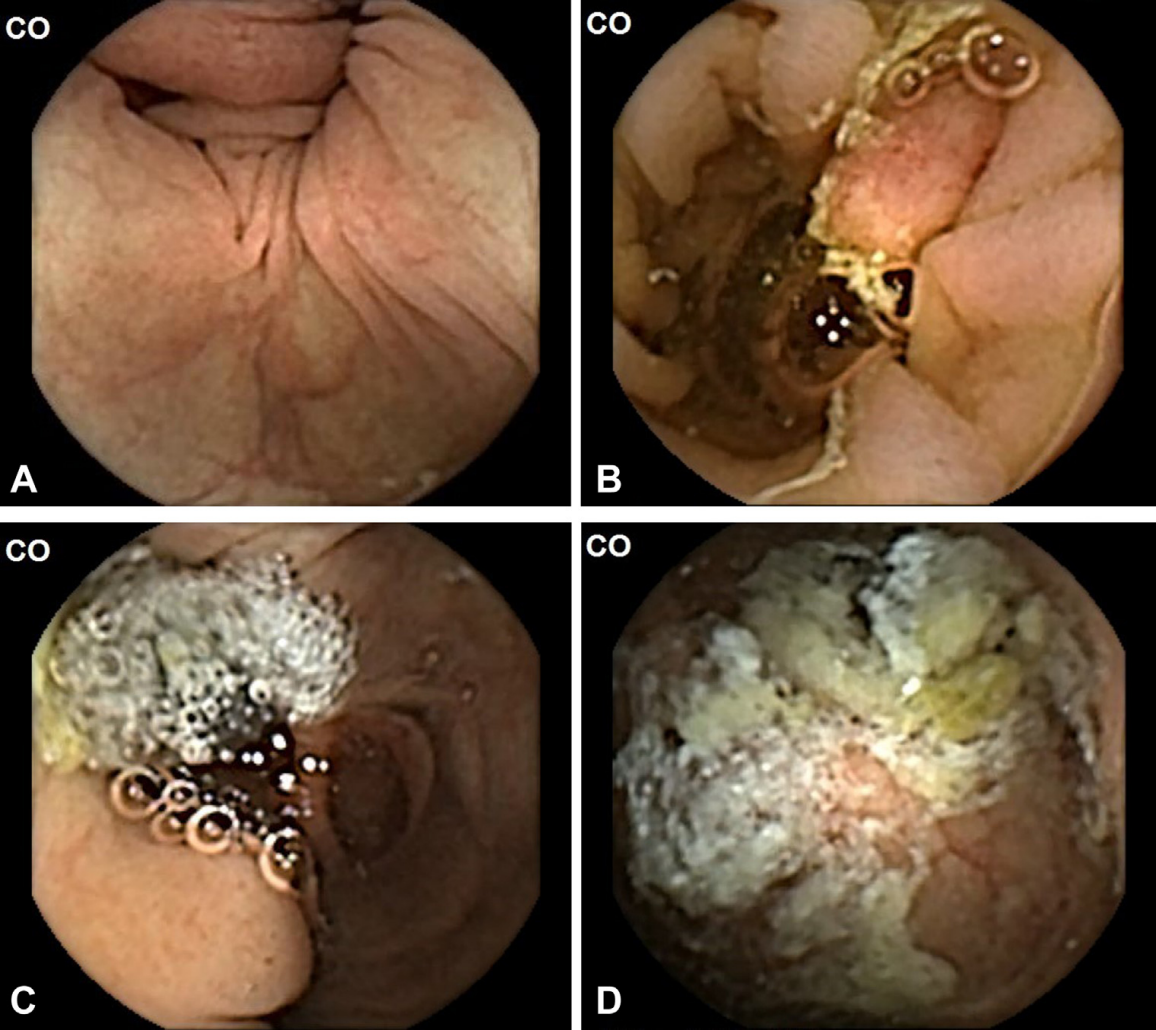
Examples of colon capsule video frames graded according to the Leighton-Rex scale. **A,** Excellent. **B,** Good. **C,** Fair. **D,** Poor.

An external partner (Corporate Health International, Hamburg, Germany) provided a service of CCE reporting by experienced CCE readers. Details regarding the exact level of experience and composition of readers were not available because the readers were employed externally. However, all CCE reports were checked, corrected, and approved by a small group of physicians with extensive experience in gastroenterology and endoscopy from the external private contractor. During these assessments, the Leighton-Rex cleansing level scale was used to assess bowel-cleansing quality on each of the 5 colonic segments.^3^ Results were reported using a scoring system with a 2-point scale (nonacceptable or acceptable bowel-cleansing quality) and a 4-point scale (poor, fair, good, and excellent) (Fig. 1). On the 2-point scale, poor bowel cleansing was considered nonacceptable, whereas fair, good, and excellent bowel cleansing were deemed acceptable.^10^ For the overall assessment to be acceptable, each of the 5 colon segments needed to be assessed as such.

### AIA: cleansing quality

**Figure 2:**
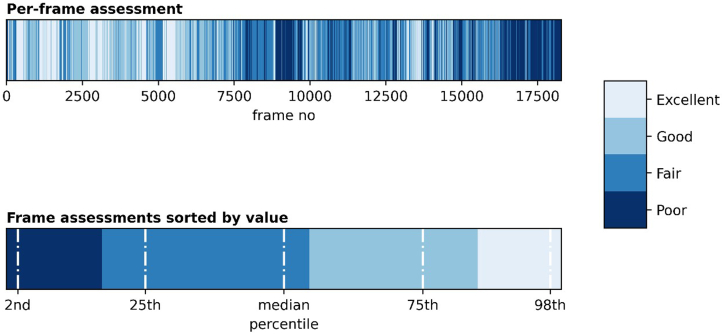
Cleansing evaluation example from the artificial intelligence algorithm.

We developed a machine learning–based AIA to evaluate bowel-cleansing quality. This algorithm determined the cleanliness on several levels. First, the CCE video material was analyzed on a single-frame level where the algorithm characterized each pixel as either “clean” or “dirty.” Subsequently, each frame was evaluated on the ratio of clean to dirty pixels. See Buijs et al^11^ for a description of the AIA and its tuning in detail. Extending on Buijs et al, we evaluated the cleanliness of video sequences based on these per-frame evaluations using the mean score, median, upper and lower quartiles, and second and 98th percentile of the distribution of frame-cleanliness ratings for each video sequence (Fig. 2). The AIA output was then mapped to the Leighton-Rex scale by rounding down to the nearest integer.

Next, the colon was divided into 3 segments determined by the hepatic and splenic flexures (ie, right-sided colon, transverse colon, and left-sided colon). The CCE reader–reported timestamps were used to identify the flexures, because the algorithm did not have localization capabilities to identify landmarks or segments. Therefore, timestamps dividing the colon into 3 segments were provided from the report. The AIA was applied, grading the overall bowel-cleansing quality and the 3 segments of the colon separately. No timestamps dividing the cecum from the ascending colon and the rectum from the sigmoid colon were available in the CCE report, and, because of this, an evaluation by the algorithm on 5 segments was not possible to give for the entire dataset.

Of 856 CCE videos, a random sample of 20 CCE videos was obtained for sensitivity analysis. This sample was reviewed by an experienced endoscopist (B.S.-O.), who divided each video into sections representing the 5 colon segments (cecum, right-sided colon, transverse colon, left-sided colon, and rectum). For the sensitivity analysis subgroup, all 5 colon segments were evaluated.

### Statistical analysis

The interobserver agreement between CCE readers and AIA was compared using Cohen’s κ for binary evaluations (ie, acceptable vs unacceptable bowel preparation) and weighted Cohen’s κ for ordinal categorical outcomes (ie, Leighton-Rex scale).^12^ First, the overall assessment of the entire colon per patient was compared. After that, per-segment comparisons were made for all segments evaluated. For these per-segment comparisons, the colon was divided into 3 segments segregated by flexures. Because the CCE readers used 5 segments, the cecum and ascending colon evaluations were combined into the right-sided colon and evaluated as the lowest scoring of the 2. The same method was used in combining the descending colon and rectum into the left-sided colon. Segmental comparisons were also performed in the subgroup, enabling us to compare colonic segments stratified by the 5 segments.

Random selection of the subgroup, data management, and statistical analyses were performed using SAS software version 9.4 (SAS Institute Inc, Cary, NC, USA). The interpretation of the level of agreement is listed in Table 1.^13^

**Table 1:** Interpretation of the level of interobserver agreement

Cohen’s κ value	Interpretation
≤.2	None
.21-.39	Minimal
.40-.59	Weak
.60-.79	Moderate
.80-.90	Strong
>.90	Almost perfect

From Ranganathan et al.^12^

### Ethics

The Regional Health Research Ethics Committee (Ref. S-20190100) and the Danish Data Protection Agency (Ref. 19/29858) approved the CareForColon2015 trial. All participants gave their written consent to participate after having received both oral and written study information.

## Results

**Figure 3:**
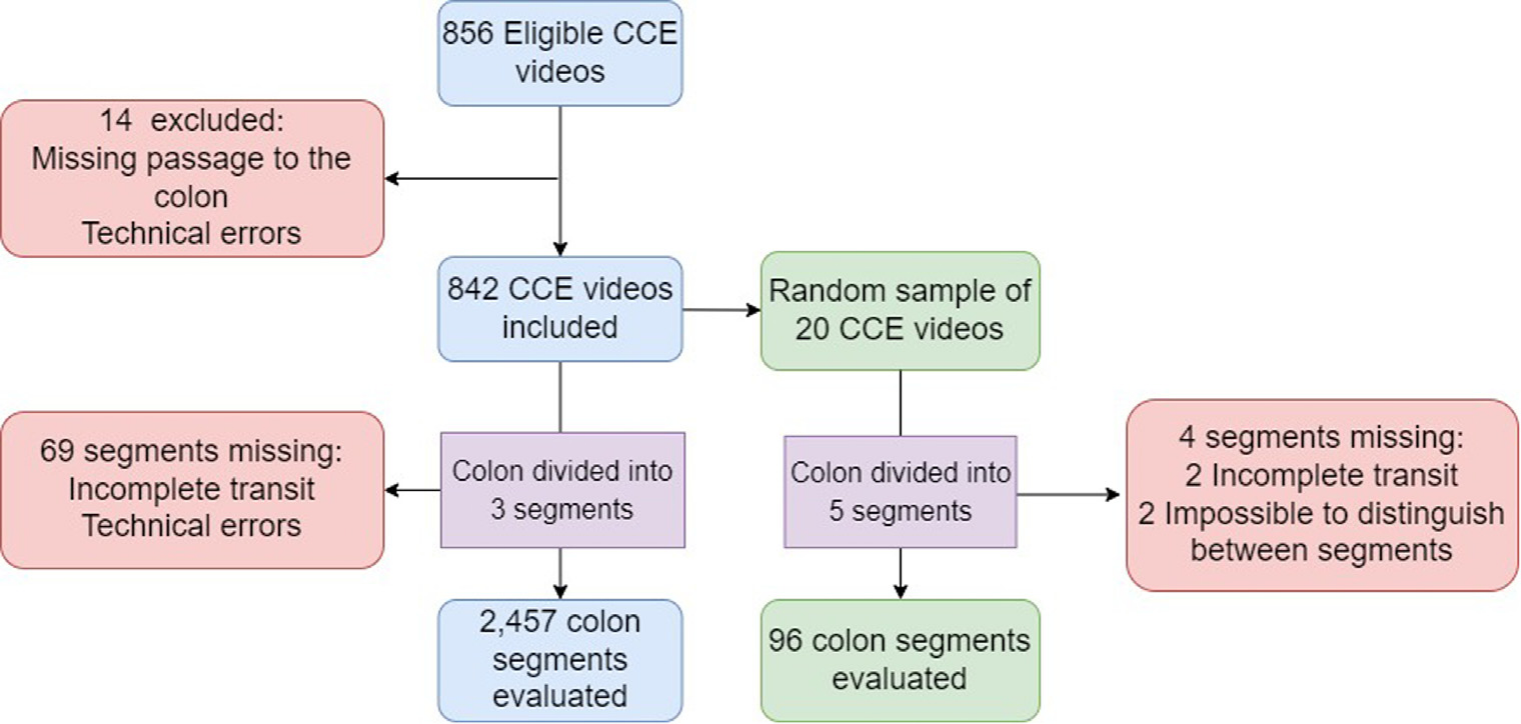
Study flowchart. *CCE*, Colon capsule endoscopy.

Of 856 consecutive CCE videos available for inclusion in our study, 14 were excluded because of missing video material of the colon (Fig. 3). The missing data were caused by instances where the capsule was retained in the stomach or small bowel or technical errors in the video-recording process. The 842 videos comprised 2457 evaluated colon segments. In the subgroup of 20 videos, 96 colon segments were evaluated. The mean age of participants was 62.6 years, and 54.3% were men.

### Overall bowel-cleansing quality

We observed a 73.9% and 73.8% agreement between CCE readers and AIA, respectively, on the 2-point grading scale (Table 2), equivalent to an interobserver agreement that was minimal to none (Cohen’s κ, .10-.12) (Table 3). In 23% of investigations, the algorithm scored the overall bowel-cleansing quality as adequate in cases where the CCE readers did not (Table 2). The agreement on the 4-point grading scale was also achieved as minimal to none (weighted κ, .07-.11) (Table 3), with 28.9% and 42.9% agreement (Supplementary Table 1, available online at www.igiejournal.org).

**Table 2:** Distribution of bowel-cleansing quality evaluation of the total colon: 2-point grading scale

		CCE readers		
		Nonacceptable BC	Acceptable BC	Total
AIA mean vs CCE readers				
AIA	Nonacceptable BC	26 (3.09)	24 (2.85)	50 (5.94)
	Acceptable BC	196 (23.28)	596 (70.78)	792 (94.06)
	Total	222 (26.37)	620 (73.63)	842 (100)
*AIA median vs CCE readers*				
AIA	Nonacceptable BC	30 (3.56)	29 (3.44)	59 (7.01)
	Acceptable BC	192 (22.80)	591 (70.19)	783 (92.99)
	Total	222 (26.37)	620 (73.63)	842 (100)

Values are n (%).

*AIA*, Artificial intelligence algorithm; *CCE*, colon capsule endoscopy; *BC*, bowel cleansing.

**Table 3:** Interobserver agreement on bowel-cleansing quality between colon capsule endoscopy readers and the artificial intelligence algorithm

	2-point grading scale Cohens κ (95% confidence interval)	4-point grading scale weighted κ (95% confidence interval)
Colon total, mean	.10 (.05-.16)	.11 (.06-.16)
Colon total, median	.12 (.05-.18)	.07 (.03-.10)
Segmental, mean	.17 (.14-.21)	.14 (.11-.17)
Segmental, second percentage	.02 (.01-.02)	.02 (.01-.02)
Segmental, 25th percentage	.09 (.08-.11)	.07 (.06-.09)
Segmental, median	.15 (.12-.17)	.12 (.10-.14)
Segmental, 75th percentage	.17 (.13-.21)	.16 (.13-.19)
Segmental, 98th percentage	.09 (.05-.14)	.06 (.04-.08)

Two-point grading scale: acceptable and nonacceptable bowel cleansing. Four-point grading scale: poor, fair, good, and excellent bowel cleansing. Colon total, 842; segmental total, 2457.

### Segmental bowel-cleansing quality

The agreement on the 2-point grading scale was minimal to none between CCE readers and the algorithm, with Cohen’s κ ranging from .02 to .17 depending on the threshold. The same was the case for the 4-point grading scale, with Cohen’s weighted κ ranging from .02 to .16 (Table 3). The AIA underestimated bowel-cleansing quality compared with the evaluation by the CCE readers in all scenarios except when using the 98th percentile (see Supplementary Table 2). Summaries on the distribution of bowel-cleansing quality evaluation on the 2- and 4-point grading scales for CCE readers and AIA mean, quartiles, and extremes are provided in Supplementary Tables 2, 3, and 4 (available online at www.igiejournal.org).

### Interobserver agreement on 5 colonic segments

In the subgroup in which we were able to compare and stratify by all 5 colonic segments, the agreements were all minimal to none, with Cohen’s κ ranging from –.20 to .38. The only exceptions to this were the weak to moderate agreement for the transverse colon using the mean AIA score (Cohen’s κ, .69; weighted κ, .52) and a perfect agreement for the rectum using the 98th percentile AIA score (all were classified as acceptable bowel-cleansing quality). All κ values for each grading scale stratified by colonic segment are provided in Supplementary Table 4.

## Discussion

In CCE, to date, manual evaluation is considered the criterion standard. However, this practice can be time-consuming, and the reported results are subject to observer variation. This creates grounds for substantial inter- and intraobserver variations on several parameters, such as cleansing quality evaluation and polyp detection.^4^ Thus far, intra- and interobserver agreements in CCE have been sparsely examined. Herein, we proposed an AIA evaluating the quality of bowel cleansing to support and ease the manual work while providing an objective and consistent result. Evaluations by CCE readers were compared with this algorithm. The interobserver agreement was minimal to none in almost all scenarios. This AIA has previously performed well compared with other models on a small sample.^11^ In the pilot study by Buijs et al,^11^ the median cleansing quality reported by the AIA was used in the analysis.

When applying AI to this field, particular challenges must be considered. For example, in CCE, the capsule moves backward and forward and can remain in the same location for an extended period. This can lead to an over-representation of specific colonic parts in the video material and thereby of the bowel-cleansing quality in this particular colonic segment or area. With this in mind during the manual assessment, the reader can, to some extent, adjust the bowel-cleansing grade. The AIA, on the other hand, creates the score based on a cleansing quality score for every frame, giving each frame the same impact. The results delivered by the AIA are directly translatable to the Leighton-Rex score, which until recently was the only scale for assessing bowel-cleansing quality in CCE.

We had hoped to find a cutoff point in good agreement with the CCE readers by analyzing the results based on different percentiles. This was not the case and may be because of the Leighton-Rex scale's qualitative nature. In other studies, the inter- and intraobserver agreements are weak to moderate.^3^^,^^4^ Therefore, the poor agreement between our AIA and the Leighton-Rex scale evaluation may not result from an inconsistent or inaccurate algorithm but from subjective human evaluation. According to a new systematic review, both intra- and interobserver agreement in capsule endoscopy reading are suboptimal.^14^ This lack of a high-quality reference standard is 1 major challenge in constructing AI solutions. A recent study comparing the Leighton-Rex scale with a new reader-dependent model, the Colon Capsule CLEansing Assessment and Report (CC-CLEAR), reported an interobserver agreement coefficient for the Leighton-Rex scale of .772 and CC-CLEAR of .911.^5^ CC-CLEAR is a quantitative scale based on the percentage of visualized mucosa. Our AIA bases its evaluation on discriminating between “clean” and “dirty” pixels. If adapting the algorithm to CC-CLEAR, we may get more consistent and objective assessments of the bowel-cleansing quality in CCE.

Another approach for improving the algorithm’s results is to adjust the thresholds in such a way that the difference to the human assessment is minimized. This adjustment should be based on a new reference standard that can be established by an expert panel consensus. A limitation of our study is the possible experience-level heterogeneity of the human CCE reader group. When a CCE is inconclusive because of nonacceptable bowel cleansing, the patient is referred to colonoscopy. With conservative evaluation of bowel-cleansing quality, a number of patients will be subjected to unnecessary colonoscopies. Conversely, a more lenient assessment of the cleansing quality can lead to missed pathology with all the relevant consequences for the individual patient and healthcare systems.

In optimizing the process for evaluating bowel-cleansing quality in CCE, we believe the subjective aspect should be minimized. Further development of the AIA presented in this study may assist us in reaching this goal. If the AIA can be adapted to create an output of the proportion of mucosa visualized, it may better agree with CCE readers but can also be compared with the new CC-CLEAR scale.

We believe that for CCE to be applicable in future clinical practice, AIAs are needed to improve reporting quality. This is not only the case for evaluation of cleansing quality, but for detection and characterization of pathology as well. In the large ongoing project, AICE (AI-supported analysis in large bowel camera Capsule Endoscopy),^15^ development of AIAs for this exact purpose is a main objective, giving us reason to hope for imminent measures to optimize CCE as a diagnostic modality.

In conclusion, overall, the interobserver agreement between CCE readers and AIA on bowel-cleansing quality was minimal. We believe the lack of a high-quality reference standard contributes to the challenges in creating a clinically useful AI solution. Furthermore, mean or percentiles of the AIA grading are not suitable for comparing the AI-generated results to the manual evaluation. The AIA may benefit from adaptations that enable an output of the proportion of mucosa visualized.

## Disclosure

The following author disclosed financial relationships: A. Koulaouzidis: Co-director and shareholder in iCERV Ltd; consultant for Jinshan Ltd and Diagmed Healthcare Ltd; travel support from Jinshan Ltd and Diagmed Healthcare Ltd; research support from ESGE/Given Imaging Ltd and IntroMedic/SynMed; travel support and speakers fees from Jinshan Ltd and consultancy fees from Medtronic; advisory board member for ANKON. All other authors disclosed no financial relationships. Sources of funding for the project supplying data for this study: Aage and Johanne Louis-Hansen’s Fond (grant 17-2B-1409), Odense University Hospital’s innovation fund (grant R75-A3392), Medtronic Research Foundation (grant ERP 2018-11151), the Danish Cancer Society (grant R100-A6747), and the Excellence Centre in the Region of Southern Denmark (grant 18/48426) for the CAREforCOLON 2015 trial; Medtronic VR (providing capsules), and B. Schelde-Olesen: received research support from the University of Southern Denmark and Odense University Hospitals PhD fund.

## Figures and Tables

**Supplementary Table 1:** Distribution of bowel-cleansing quality evaluation of the total colon: Leighton-Rex 4-point grading scale

		CCE readers				
		Poor	Fair	Good	Excellent	Total
AIA median vs CCE readers						
AIA	Poor	30 (3.6)	24 (2.8)	4 (.5)	1 (.1)	59 (7.0)
	Fair	102 (12.1)	146 (17.3)	73 (8.7)	1 (.1)	322 (38.2)
	Good	61 (7.2)	154 (18.3)	66 (7.8)	3 (.4)	284 (33.7)
	Excellent	29 (3.4)	88 (10.5)	58 (6.9)	2 (.2)	177 (21.0)
	Total	222 (26.4)	412 (48.9)	201 (23.9)	7 (.8)	842 (100)
*AIA mean vs CCE readers*						
AIA	Poor	26 (3.1)	20 (2.4)	3 (.4)	1 (.1)	50 (5.9)
	Fair	151 (17.9)	252 (29.9)	115 (13.7)	1 (.1)	519 (61.6)
	Good	45 (5.3)	140 (16.6)	83 (9.9)	5 (.6)	273 (32.4)
	Excellent	0 (.0)	0 (.0)	0 (.0)	0 (.0)	0 (.0)
	Total	222 (26.4)	412 (48.9)	201 (23.9)	7 (.8)	842 (100)

Values are n (%).

*AIA*, Artificial intelligence algorithm; *CCE*, colon capsule endoscopy.

**Supplementary Table 2:** Colon segmental distribution of bowel-cleansing quality evaluation: Leighton-Rex 4-point grading scale

		CCE readers				
		Poor	Fair	Good	Excellent	Total
AIA mean vs CCE readers						
AIA	Poor	238 (9.69)	433 (17.62)	274 (11.15)	4 (.16)	949 (38.62)
	Fair	106 (4.31)	410 (16.69)	425 (17.30)	14 (.57)	955 (38.87)
	Good	41 (1.67)	221 (8.99)	273 (11.11)	16 (.65)	551 (22.43)
	Excellent	0 (.00)	0 (.00)	2 (.08)	0 (.00)	2 (.08)
	Total	385 (15.67)	1064 (43.30)	974 (39.64)	34 (1.38)	2457 (100)
*AIA second percentile vs CCE readers*						
AIA	Poor	374 (15.22)	1005 (40.90)	883 (35.94)	22 (.90)	2284 (92.96)
	Fair	6 (.24)	46 (1.87)	67 (2.73)	7 (.28)	126 (5.13)
	Good	2 (.08)	7 (.28)	18 (.73)	5 (.20)	32 (1.30)
	Excellent	3 (.12)	6 (.24)	6 (.24)	0 (.00)	15 (.61)
	Total	385 (15.67)	1064 (43.30)	974 (39.64)	34 (1.38)	2457 (100)
*AIA median vs CCE readers*						
AIA	Poor	304 (12.37)	614 (24.99)	402 (16.36)	12 (.49)	1332 (54.21)
	Fair	58 (2.36)	309 (12.58)	388 (15.79)	8 (.33)	763 (31.05)
	Good	13 (.53)	104 (4.23)	121 (4.92)	5 (.20)	243 (9.89)
	Excellent	10 (.41)	37 (1.51)	63 (2.56)	9 (.37)	119 (4.84)
	Total	385 (15.67)	1064 (43.30)	974 (39.64)	34 (1.38)	2457 (100)
*AIA 98th percentile vs CCE readers*						
AIA	Poor	51 (2.08)	82 (3.34)	45 (1.83)	1 (.04)	179 (7.29)
	Fair	88 (3.58)	169 (6.88)	112 (4.56)	7 (.28)	376 (15.30)
	Good	126 (5.13)	318 (12.94)	255 (10.38)	4 (.16)	703 (28.61)
	Excellent	120 (4.88)	495 (20.15)	562 (22.87)	22 (.90)	1199 (48.80)
	Total	385 (15.67)	1064 (43.30)	974 (39.64)	34 (1.38)	2457 (100)

Values are n (%).

*AIA*, Artificial intelligence algorithm; *CCE*, colon capsule endoscopy.

**Supplementary Table 3:** Colon segmental distribution of bowel-cleansing quality evaluation: 2-point grading scale

		CCE readers		
		Nonacceptable BC	Acceptable BC	Total
*AIA mean vs CCE readers*				
AIA	Nonacceptable BC	238 (9.69)	711 (28.94)	949 (38.62)
	Acceptable BC	147 (5.98)	1361 (55.39)	1508 (61.38)
	Total	385 (15.67)	2072 (84.33)	2457 (100)
*AIA second percentile vs CCE readers*				
AIA	Nonacceptable BC	374 (15.22)	1910 (77.74)	2284 (92.96)
	Acceptable BC	11 (.45)	162 (6.59)	173 (7.04)
	Total	385 (15.67)	2072 (84.33)	2457 (100)
*AIA median vs CCE readers*				
AIA	Nonacceptable BC	304 (12.37)	1028 (41.84)	1332 (54.21)
	Acceptable BC	81 (3.30)	1044 (42.49)	1125 (45.79)
	Total	385 (15.67)	2072 (84.33)	2457 (100)
*AIA 98th percentile vs CCE readers*				
AIA	Nonacceptable BC	51 (2.08)	128 (5.21)	179 (7.29)
	Acceptable BC	334 (13.59)	1944 (79.12)	2278 (92.71)
	Total	385 (15.67)	2072 (84.33)	2457 (100)

Values are n (%).

*AIA*, Artificial intelligence algorithm; *CCE*, colon capsule endoscopy; *BC*, bowel cleansing.

**Supplementary Table 4:** Subgroup: interobserver agreement on bowel-cleansing quality between CCE readers and the AI algorithm

	2-point grading scale Cohens κ (95% confidence interval)	4-point grading scale weighted κ (95% confidence interval)
Cecum, mean	.15 (–.06 to .35)	.12 (–.03 to .26)
Cecum, second percentile	.05 (–.04 to .13)	.07 (–.03 to .17)
Cecum, 25th percentile	.07 (–.04 to .18)	.08 (–.02 to .18)
Cecum, median	.15 (–.06 to .35)	.11 (–.02 to .25)
Cecum, 75th percentile	.22 (–.06 to .51)	.17 (.01-.35)
Cecum, 98th percentile	.15 (–.31 to .61)	.14 (–.09 to .37)
AC, mean	.13 (–.05 to .31)	.09 (–.10 to .27)
AC, second percentile	.06 (–.04 to .16)	.06 (–.03 to .14)
AC, 25th percentile	.13 (–.05 to .31)	.13 (–.01 to .27)
AC, median	.16 (–.06 to .37)	.09 (–.08 to .27)
AC, 75th percentile	–.20 (–.45 to .05)	–.02 (–.17 to .13)
AC, 98th percentile	–.12 (–.23 to .00)	–.15 (–.34 to .04)
TC, mean	.69 (.31-1.0)	.52 (.24-.80)
TC, second percentile	.08 (–.03 to .20)	.08 (–.03 to .20)
TC, 25th percentile	.25 (–.01 to .52)	.30 (.07-.54)
TC, median	.38 (–.09 to .86)	.34 (.13-.55)
TC, 75th percentile	–.08 (–.21 to .04)	.11 (–.06 to .29)
TC, 98th percentile	NA, AI evaluated all acceptable	.04 (–.04 to .11)
DC, mean	–.08 (–.21 to .04)	–.09 (–.41 to .22)
DC, second percentile	.17 (–.03 to .38)	.14 (–.01 to .29)
DC, 25th percentile	.08 (–.37 to .53)	–.03 (–.32 to .27)
DC, median	–.08 (–.21 to .04)	–.01 (–.18 to .16)
DC, 75th percentile	–.08 (–.21 to .04)	–.04 (–.13 to .04)
DC, 98th percentile	NA, AI evaluated all acceptable	NA, AI evaluated all good or excellent
Rectum, mean	NA, CCE readers evaluated all acceptable	.06 (–.23 to .36)
Rectum, second percentile	NA, CCE readers evaluated all acceptable	.06 (–.04 to .16)
Rectum, 25th percentile	NA, CCE readers evaluated all acceptable	.18 (–.04 to .40)
Rectum, median	NA, CCE readers evaluated all acceptable	–.01 (–.30 to .28)
Rectum, 75th percentile	NA, CCE readers evaluated all acceptable	–.06 (–.34 to .22)
Rectum, 98th percentile	Perfect agreement	.08 (–.07 to .23)

Two-point grading scale: acceptable and nonacceptable bowel cleansing. Four-point grading scale: poor, fair, good, and excellent bowel cleansing. Cecum, 18; ascending colon, 19; transverse colon, 20; descending colon, 20; rectum, 19.

*CCE*, Colon capsule endoscopy; *AI*, artificial intelligence; *AC*, ascending colon; *TC*, transverse colon; *DC*, descending colon; *NA*, not applicable.
